# Pediatric injury prevention: emerging epidemics within a pandemic

**DOI:** 10.1186/s40621-022-00406-5

**Published:** 2022-12-21

**Authors:** Michael N. Levas

**Affiliations:** Pediatrics, Emergency Medicine, Medical College of Wisconsin, Milwaukee, USA

As the world pushes forward through the COVID-19 pandemic, it has become apparent that many in our society disproportionately carry the burdens of strains on our healthcare system. From an increased risk of morbidity and mortality (Magesh et al. 2021) to decreased healthcare access (Moynihan et al. 2021), our marginalized populations suffered disproportionately. Injury prevention and science was not immune to these burdens and stressors. In fact, one could argue that many pediatric institutions had to face new epidemics of injury while navigating the impacts of COVID-19.

During the COVID-19 pandemic, many of our beliefs about risk and injury were challenged. For the first time, firearms became the leading cause of death in youth, surpassing motor vehicle crashes (Lee et al. 2022). Drug overdoses and suicide rates changed (Faust et al. 2021). Funding and workforce changes challenged many injury prevention programs across the country. For 27 years, the Injury Free Coalition for Kids ® has continued to be one of the most impactful injury prevention organizations in the country. Despite the stressors on healthcare systems, our membership blossomed to 48 institutions, each dedicated to continued research, education, and advocacy in the field of pediatric injury prevention.

In this issue, the Injury Free Coalition for Kids® annual meeting supplement in Injury Epidemiology, we highlight the continued pediatric injury prevention efforts occurring nationally. Given the impact of COVID-19, studies evaluate changes in parental home safety practices before and during the pandemic and changes in injury presentations to the pediatric emergency department. This issue further explores the disparities caused or exacerbated during the pandemic through the examination of parental attitudes toward firearm storage, the impact of firearm prevention efforts in suicidal youth, and descriptions of differential reporting based on race in sudden unexpected infant death (SUID). Many pediatric injury prevention efforts and research endured through the pandemic with topics ranging from All-Terrain Vehicle Safety to poisoning in youth with autism spectrum disorder, and their efforts are highlighted in this issue.

Injury Free Coalition for Kids® endures because our member hospitals, clinicians, and injury prevention specialists know that only through adequate knowledge and advocacy can we prevent childhood injury and death. We believe that shared knowledge about best practices is the way forward in avoiding preventable morbidity and mortality caused by pediatric injury. The studies in this issue are only a fraction of the work being done throughout the Injury Free Coalition for Kids®. Please enjoy these scholarly articles. Hopefully, they encourage your efforts to join us in making our youth Injury Free.

## Data Availability

Not applicable.
